# Acid excretion is impaired in calcium oxalate stone formers

**DOI:** 10.1093/ndt/gfaf038

**Published:** 2025-02-20

**Authors:** Pedro H Imenez Silva, Nasser A Dhayat, Daniel G Fuster, Harald Seeger, Alexander Ritter, Thomas Ernandez, Florian Buchkremer, Beat Roth, Olivier Bonny, Isabel Rubio-Aliaga, Carsten A Wagner

**Affiliations:** Institute of Physiology, University of Zurich, Zurich, Switzerland; National Center of Competence of Research NCCR Kidney.CH, Switzerland; Department of Internal Medicine, Division of Nephrology and Transplantation, Erasmus Medical Center, University Medical Center, Rotterdam, The Netherlands; Nephrology and Dialysis Care Center, B. Braun Medical Care AG, Hochfelden, Switzerland; Department of Nephrology and Hypertension, Inselspital, Bern University Hospital, University of Bern, Bern, Switzerland; Division of Nephrology, University Hospital Zurich, Zurich, Switzerland; Institute for Nephrology and Dialysis, Cantonal Hospital Baden, Baden, Switzerland; Clinic for Nephrology and Transplantation Medicine, Cantonal Hospital St. Gallen, St. Gallen, Switzerland; Division of Nephrology, Kantonsspital Aarau, Aarau, Switzerland; Division of Nephrology, Kantonsspital Aarau, Aarau, Switzerland; Department of Urology, Inselspital, Bern University Hospital, University of Bern, Bern, Switzerland; Service of Nephrology, Fribourg State Hospital and University of Fribourg, Fribourg, Switzerland; National Center of Competence of Research NCCR Kidney.CH, Switzerland; Service of Nephrology, Lausanne University Hospital, Lausanne, Switzerland; Institute of Physiology, University of Zurich, Zurich, Switzerland; National Center of Competence of Research NCCR Kidney.CH, Switzerland; Institute of Physiology, University of Zurich, Zurich, Switzerland; National Center of Competence of Research NCCR Kidney.CH, Switzerland

To the Editor,

Urine pH and supersaturation contribute to urine crystal formation [1]. However, how the renal capacity to excrete acid influences the formation of common kidney stone types is not completely understood. Leveraging the extensive urine biochemistry profile available in the Swiss Kidney Stone Cohort (SKSC) [2], we tested whether urinary acid–base (AB) parameters, including pH, ammonium, titratable acidity (TA), net acid excretion (NAE) and citrate, were associated with the presence of calcium oxalate (CaOx) and calcium phosphate (CaP) stones. We assessed the kidney's capacity of excreting acids by calculating an index that determines how much net acid is excreted in relation to urine pH, which was previously termed as net acid excretion capacity (NAEC) [3]. The NAEC is obtained from the residuals of the relation between net acid excretion and urine pH. A similar ammonium-to-pH index, the AB score, was recently validated in chronic kidney disease [4]. We also included titratable acid and gastrointestinal alkali absorption in our analyses as similarly done in Ferraro *et al.* [5].

The SKSC is a prospective, multicentric, longitudinal cohort of patients with kidney stones [2]. Participants with kidney stone disease [stone formers (SFs)] were followed for 3 years to collect blood, urine samples and clinical data. This analysis used baseline data collected 2 weeks after screening, with the last stone episode occurring 6–17 weeks prior to baseline (Supplementary Tables S1–S3). Stone composition was determined via Fourier transform infrared spectroscopy. A control group [non–stone formers (NSFs)], confirmed by computed tomography (CT) to be free of kidney stones and calcifications, was also recruited. Exclusion criteria specific to this study are in Supplementary Fig. S1. Biochemical parameters were measured as described in Bonny *et al.* [2], with participants eating their usual diet, and data analysis was conducted using R/RStudio (Posit, Boston, MA, USA) [6].

We included 309 CaOx and 28 CaP SFs and 193 NSFs in this study. We tested whether urinary AB parameters were associated with the presence of CaOx or CaP stones in these individuals. We performed logistic regression models using Z-scores for urine ammonium, pH, citrate, phosphate, calcium, magnesium and oxalate as independent variables (model 0) and also corrected this model using age, body mass index (BMI) and sex (model 1). Linearity between continuous covariates and the dependent variable was assessed via a component residual plot. Multicollinearity was evaluated by calculating the variance inflation factor (VIF), which ranged from 1.09 to 2.05, indicating low to moderate multicollinearity. Nevertheless, some degree of multicollinearity is inherent in our analysis. According to model 0 for CaOx, holding all other independent variables constant, urine ammonium, pH and citrate were negatively and calcium was positively associated with the odds of having kidney stones (Supplementary Table S4). Results remained consistent after adjusting for age, BMI and sex (Supplementary Table S5). Logistic models performed poorly for CaP data and results were not included.

Urine AB parameters varied significantly among the groups (Fig. 1A). The NAEC was reduced in CaOx SFs (−1.74 ± 17.78 mEq/24 h) in relation to NSFs (3.80 ± 17.87; *P* = .002). CaP SFs showed values that were intermediate between the other two groups (0.63 ± 20.84; *P* = 1.0) (Fig. 1B). Calculated titratable acidity was similar across all groups (Fig. 1C). The AB score was reduced in CaOx SFs compared with NSFs (20.70 ± 6.28 arbitrary units versus 23.30 ± 6.63; *P* < .001) (Fig. 1D). Next, we performed a logistic regression analysis for the presence of CaOx stones, replacing urine ammonium, pH and phosphate with NAEC or ammonium and pH with AB score. The NAEC and AB score associated with CaOx stone presence {NAEC: odds ratio [OR] 0.56 [95% confidence interval (CI) 0.43–0.73], *P* < .001; AB score: OR 0.68 [95% CI 0.55–0.84], *P* < .001} and both parameters remained associated with the presence of CaOx after adjustment for age, BMI and sex (Table 1 and Supplementary Tables S6–S8).

**Figure 1:**
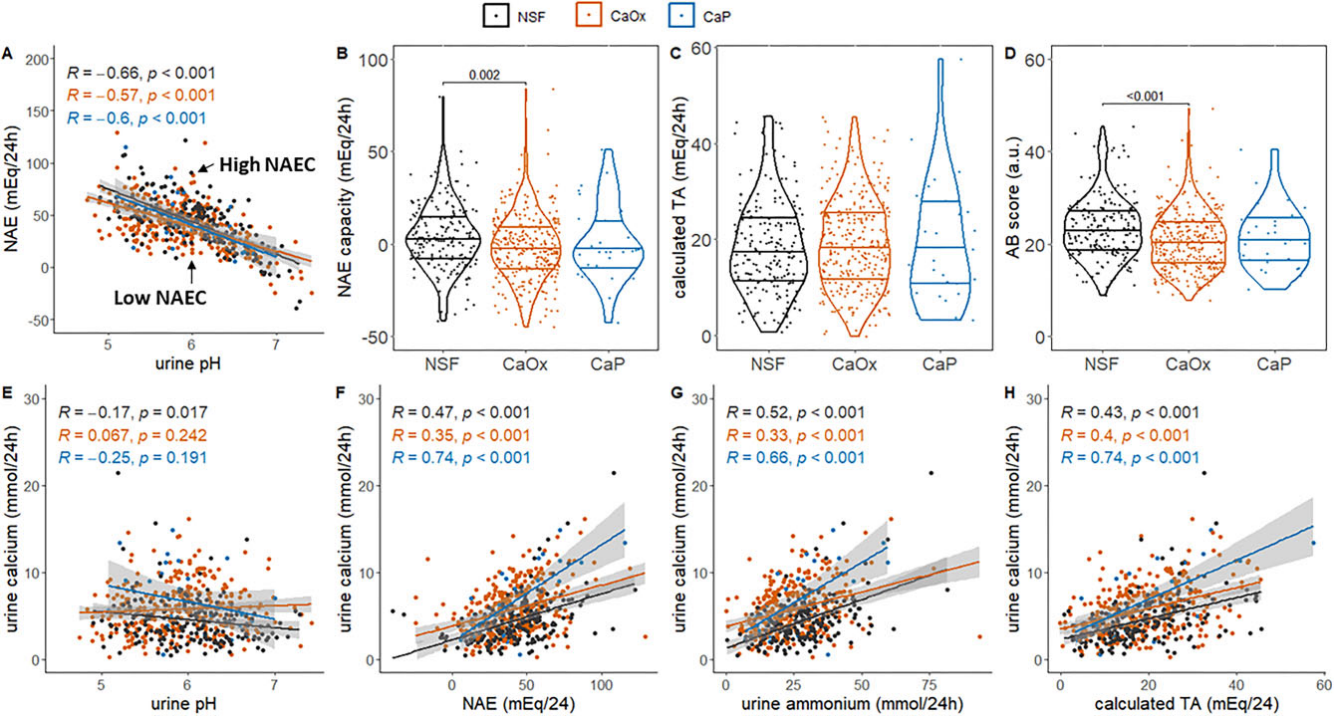
NAEC is altered in CaOx SFs and acid-dependent calcium excretion is exacerbated in CaP SFs. **(A)** Pearson correlation analysis between urine pH and NAE. **(B–D)** Violin plots showing median and 25th and 75th percentiles of (B) NAE capacity, (C) calculated TA and (D) AB score. **(E–H)** Pearson correlation analysis between (E) urine pH and urine calcium, (F) NAE and urine calcium, (G) urine ammonium and calcium and (H) calculated TA and urine calcium. Blue dots: NSFs; orange dots: CaOx SFs; black dots: CaP SFs; *R*: Pearson’s correlation coefficient and *P*-value associated with this correlation. α = 0.05.

**Table 1:** Logistic regression model 0 with CaOx stone formation as a dependent variable and NAEC replacing urine ammonium, pH and phosphate.

Urine parameter	Estimate	Standard error	Statistic	*P*-value	OR	2.50%	97.50%
(Intercept)	0.59	0.11	5.60	<.001	1.80	1.48	2.22
NAEC	−0.58	0.14	−4.29	<.001	0.56	0.43	0.73
Citrate	−0.49	0.11	−4.24	<.001	0.62	0.49	0.77
Calcium	1.07	0.15	6.94	<.001	2.93	2.18	4.00
Magnesium	−0.21	0.14	−1.50	.134	0.81	0.61	1.07
Oxalate	−0.27	0.15	−1.80	.073	0.76	0.56	1.01

The *P*-values were obtained from a Wald test with α = 0.05.

We repeated the Pearson correlation analysis and logistic models, excluding individuals who reported using supplements or medications that may affect AB balance (Supplementary Figs. S2 and S3 and Supplementary Tables S9–S15). Results were largely consistent with those shown in Fig. 1, suggesting that the pathomechanisms underlying calcium stone formation reported here may be intrinsic to the tubular function of these patients.

Worcester *et al.* [7] suggested that female CaOx SFs have reduced intestinal alkali absorption, leading to acidic urine pH. Consistent with this, we found that both alkali absorption and urine pH were reduced in CaOx SFs (Supplementary Tables S1 and S3). However, the relation of alkali absorption with urinary AB parameters and urine calcium was not significantly altered in SFs (Supplementary Figure S4). Intestinal alkali absorption showed a weak association with CaOx stone presence in logistic models (Supplementary Tables S16 and S17), but this association completely vanished if either urine pH or ammonium was removed from the model. The reduced urine pH, caused by low gastrointestinal alkali absorption (or other causes) with preserved renal proton secretion, accompanied by proportionally low NAE in CaOx SFs, may suggest a proximal tubule defect. While causality is not demonstrated, we hypothesize that impaired proximal tubule acid excretion may contribute to increased CaOx crystal deposition, consistent with our previous study in mice [8].

Last, healthy kidneys increase calcium excretion in response to an acid load and calcium has been reported to be associated with urine pH and NAE [9]. The correlation between calcium excretion and urine pH was not strong, but was still significant in NSF individuals (*R* = −0.17, *P* = .017) and it was absent in CaOx SFs (*R* = −0.07, *P* = .24) and CaP SFs (*R* = −0.25, *P* = .19) (Fig. 1E). Urine calcium was correlated with urine NAE, ammonium and calculated TA in all three groups, but these correlations were stronger and steeper in CaP SFs in relation to NSFs (Fig. 1F–H).

The main limitations of our study are the small sample size for CaP SFs, which may have prevented the detection of relevant associations between the presence of CaP stones and AB parameters, the lack of a controlled diet in our studies and the use of calculated instead of measured TA and bicarbonate. However, in the context of this study, calculated has an advantage over measured TA, as it is not affected by the precipitation of calcium phosphate [10]. Significant strengths include extensive urinary biochemical data near stone diagnosis, CT validation of our control group and detailed medication histories.

In summary, our results suggest that a reduction in urine pH not followed by proportional excretion of net acid excretion could either facilitate or be a consequence of CaOx stone deposition. The kidney's capacity for excreting acid and its interaction with calcium excretion are differentially impaired in CaP and CaOx SFs, which most probably reflects different tubular defects involved in stone formation.

## Supplementary Material

gfaf038_Supplemental_File

## Data Availability

Data underlying this research are made available upon reasonable request and considering ethical limitations.
