# Supplementary material for: Acid excretion is impaired in calcium oxalate stone formers
Source: Nephrol Dial Transplant. 2025 Feb 20;40(7):1433–5. doi: 10.1093/ndt/gfaf038 (PMC12207603; doi:10.1093/ndt/gfaf038)
Supplement: gfaf038_Supplemental_File [file gfaf038_supplemental_file.pdf]

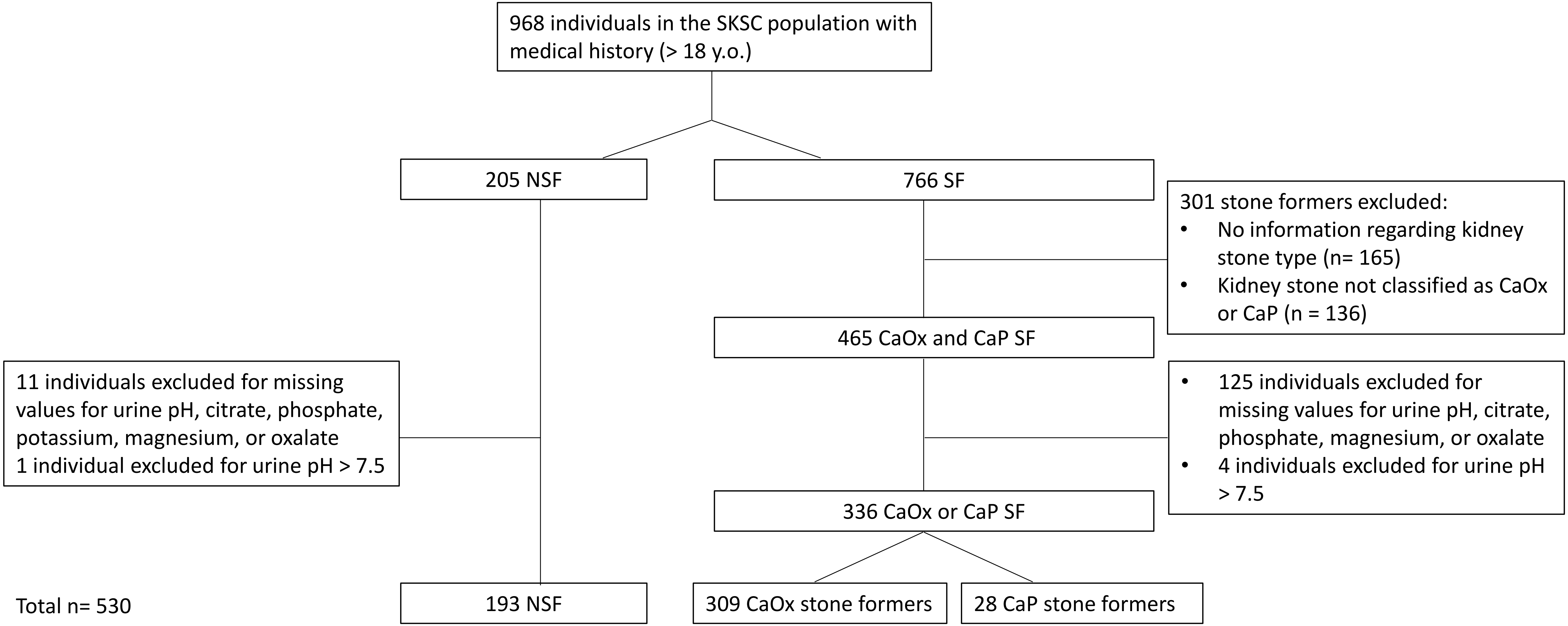

Supplementary Figure 1: Flow chart of the studied population derived from the Swiss Kidney Stone Cohort. CaOx = calcium oxalate, CaP = calcium phosphate, SF = stone former, and NSF = non-stone former.

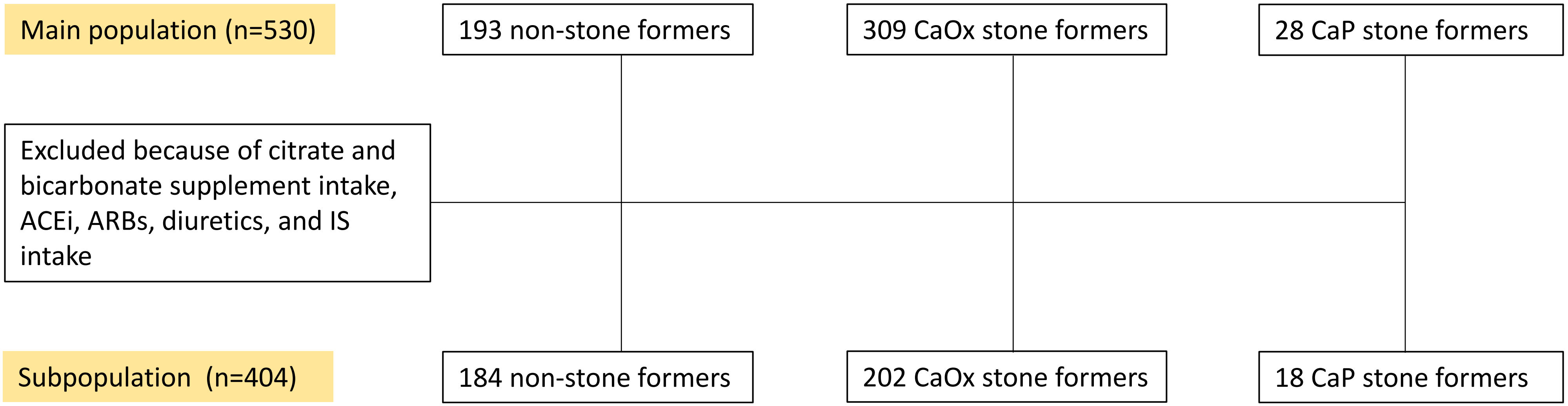

Supplementary Figure 2: Number of individuals per group after exclusion by drugs that affect the acid-base status. ACEi = Angiotensin-converting enzyme inhibitors, ARBs = Angiotensin receptor blockers, IS = immunosuppressants

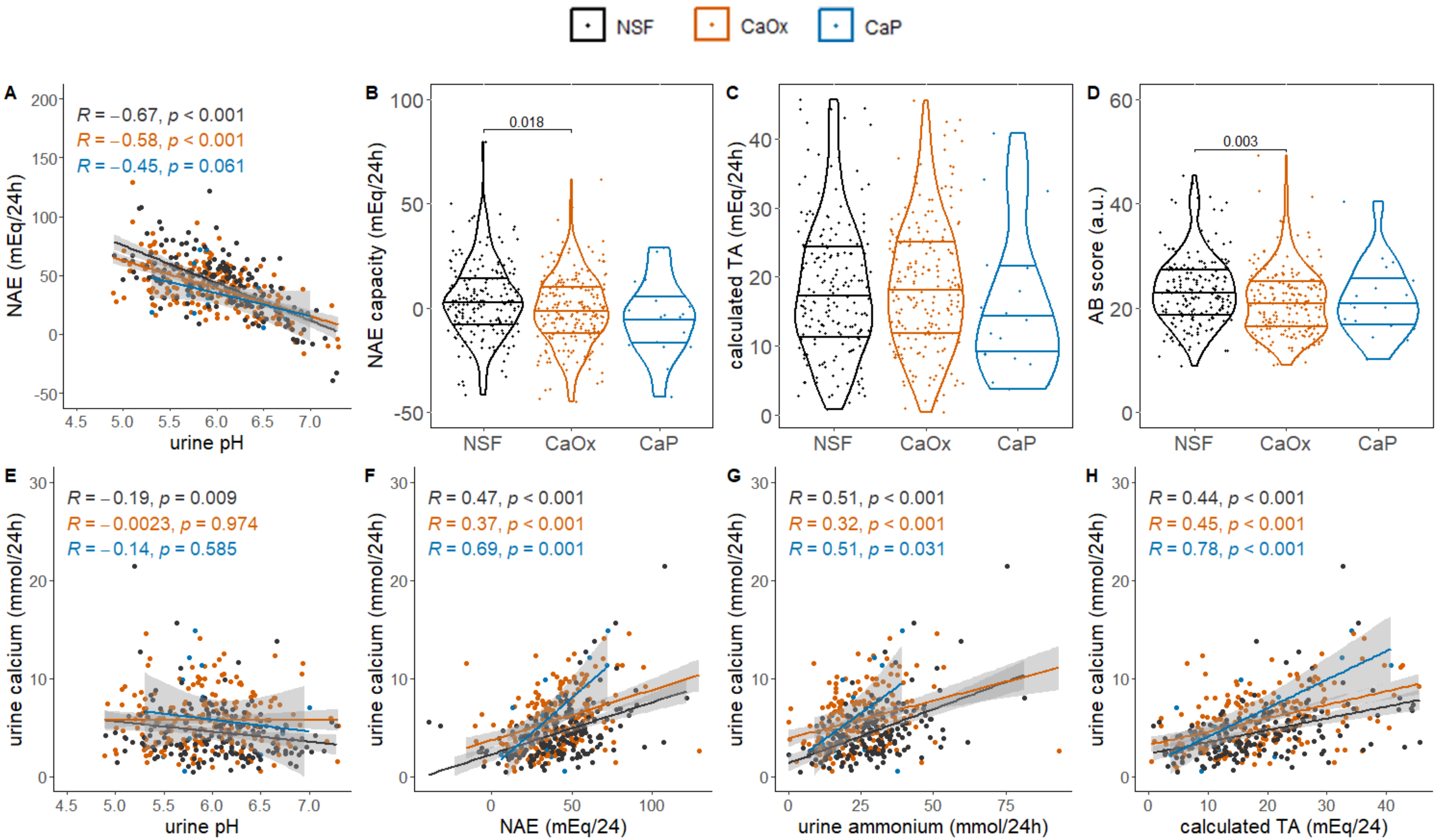

Supplementary Figure 3: Intake of medication and supplements interacting with acid-base balance did not play a major role in acid excretion capacity and its relationship with calcium excretion. (A) Pearson correlation analysis between urine pH and net acid excretion (NAE). (B-D) Violin plots showing median and 25th and 75th percentile of (B) NAE capacity, (C) calculated titratable acidity (TA), and (D) acid-base (AB) score. (E-H) Pearson correlation analysis between urine pH and urine calcium (E), NAE and urine calcium (F), urine ammonium and calcium (G), and calculated TA and urine calcium (H). Blue dots = non stone formers (NSF), orange dots = CaOx stone formers (SF), black dots = CaP SF. R = Pearson correlation coefficient and p = p-value associated with this correlation. α = 0.05.

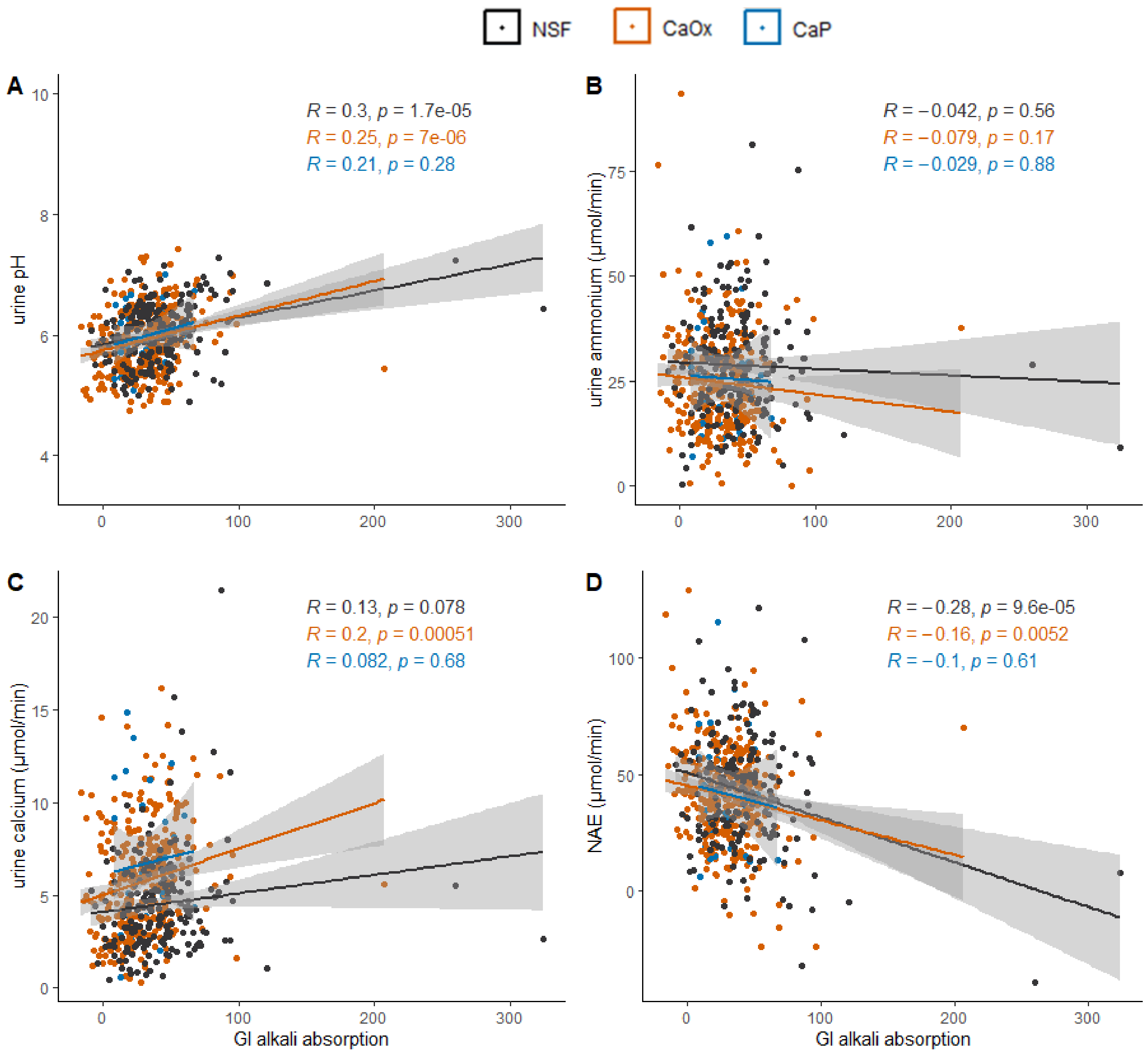

Supplementary Figure 4: Variation in gastrointestinal alkali absorption does not explain altered ammonium or calcium excretion. Correlation between calculated gastrointestinal alkali absorption and urine pH (A), ammonium (B), calcium (C), and net acid excretion is not significantly modified in CaOx or CaP stone formers. Black dots = NSF, orange dots = CaOx stone formers, and blue dots = CaP stone formers. R = Pearson correlation coefficient and p = p-value associated with this correlation. α = 0.05.

Supplementary Tables

Baseline characteristics			
	NSF (N=193)	CaOx (N=309)	CaP (N=28)
Sex (Female)	84 (43.5%)	89 (28.8%)	18 (64.3%)
Age (years)	43.0 ± 13.6	46.4 ± 14.3	44.3 ± 14.4
BMI (kg/m^2)	25.0 ± 4.12	26.9 ± 4.66	26.4 ± 5.73
Weight (kg)	75.7 ± 15.1	80.7 ± 16.4	74.7 ± 21.2
eGFR by CKD-EPI 2009 w/o race coefficient (ml/min per 1.73m2)	97.3 ± 16.1	96.8 ± 18.5	98.9 ± 16.8
GI alkali absorption (mEq/min)	29.6 ± 23.7	20.8 ± 15.6	21.0 ± 11.8
Hypertension	18 (9.4%), n =192	69 (22.3%), n = 307	9 (32.1%)
Diabetes type 1	0 (0%), n = 192	1 (0.3%), n = 305	0 (0.0%)
type 2	3 (1.6%), n = 192	28 (9.1%), 305	2 (7.1%)
Dyslipidemia	22 (11.5%), n = 192	41 (13.3%), n = 258	1 (3.6%), n = 25
Gout	2 (1.0%), n = 192	6 (1.9%), n = 305	1 (3.6%)
Smoker	43 (22.4%), n = 190	85 (27.6%), n = 301	5 (17.9%)
Ex-smoker	26 (13.5%), n = 190	38 (12.3%), n = 301	2 (7.1%)
Osteoporosis	5 (2.6%), n = 189	8 (2.6%), n = 232	0 (0.0%), n = 22
Fractures	96 (50.0%), n = 191	75 (24.3%), n = 296	4 (14.3%), n = 26
Immobilization	14 (7.3%), n = 180	15 (4.9%), n = 249	3 (10.7%), n = 23
Kidney stones in the family	29 (15.1%), n = 187	144 (46.4%), n = 305	14 (50.0%), n = 27

Kidney transplantation	1 (0.5%), n = 185	0 (0%), n = 302	1 (3.6%)
Pyelonephritis in the past	7 (3.6%)	48 (15.5%)	11 (39.3%)
Nephrocalcinosis	0 (0%), n = 191	19 (6.1%), n = 256	3 (10.7%), n = 25
Inflammatory bowel diseases	2 (1.0%), n = 189	17 (5.5%), n = 304	2 (7.1%)
Gastric bypass	2 (1.0%), n = 190	20 (6.5%), n = 304	0 (0.0%)
Vegetarian	16 (8.3%)	10 (3.2%)	1 (3.6%)
Lactose-free	9 (4.7%)	8 (2.6%)	0 (0%)
Vegan	2 (1.0%)	0 (0%)	0 (0%)
Other diets	15 (7.8%)	10 (3.2%)	3 (10.7%)
Supplements and vitamins	64 (33.3%), n =188	67 (21.7%), n = 250	8 (28.6%), n = 21

Supplementary Table 1. Baseline characteristics. Categorical data are reported as count and percentage in relation to each group's total sample size and continuous data as mean ± SD. Parameters with missing data have their sample size (n) shown next to the mean or median value of each group. GI = gastrointestinal.

Blood parameters			
	NSF (N=193)	CaOx (N=309)	CaP (N=28)
Creatinine (mmol/l)	76.4 ± 13.5, n = 192	77.5 ± 17.6, n = 308	69.9 ± 17.9, n = 27
pH	7.38 ± 0.031, n = 71	7.40 ± 0.03, n = 68	7.41 ± 0.03, n = 7
PaCO2 (mmHg)	43.6 ± 6.45, n = 100	43.6 ± 6.32, n = 113	38.1 ± 7.58, n = 10
Chloride (mmol/l)	104 ± 2.47	103 ± 2.81, n = 307	103 ± 2.0
Potassium (mmol/l)	3.99 ± 0.266	4.09 ± 0.311	3.97 ± 0.356
Sodium (mmol/l)	141 ± 2.05	141 ± 2.14	141 ± 2.20
Inorganic phosphate (mmol/l)	1.01 ± 0.160	0.98 ± 0.18	1.03 ± 0.21
Magnesium (mmol/l)	0.81 ± 0.05	0.82 ± 0.23	0.80 ± 0.05
PTH (ng/l)	37.1 ± 15.6	42.1 ± 24.2	41.9 ± 21.4, n = 27
Calcidiol (mmol/l)	57.0 [40.0, 75.0]	52.0 [34.2, 71.0]	49.5 [29.8, 70.3]
Calcitriol (mmol/l)	116 ± 33.1	118 ± 37.4	124 ± 45.0
Ionized calcium (mmol/l)	1.19 ± 0.12, = 99	1.21 ± 0.18, n = 118	1.19 ± 0.04, n = 10
FGF23 (pg/ml)	43.7 ± 13.5, n = 191	44.6 ± 25.6, n = 304	45.0 ± 15.4, n = 26
Cholesterol (mmol/l)	4.79 ± 1.01	4.82 ± 1.09	4.48 ± 0.81, n = 27
HDL (mmol/l)	1.42 ± 0.37	1.30 ± 0.37, n = 175	1.39 ± 0.34, n = 14
LDL (mmol/l)	3.15 ± 0.96	3.10 ± 1.05, n = 175	2.84 ± 0.89, n = 14

Supplementary Table 2: Blood parameters in stone former individuals and healthy NSFs. Continuous data are shown as mean ± SD and continuous skewed data as median [25th percentile; 75th percentile]. Parameters with missing data have their sample size (n) shown next to the mean or median value of each group. PTH = parathyroid hormone, LDL = low-density lipoprotein, HDL = high-density lipoprotein, FGF23 = fibroblast growth factor 23. Student t-test was used for continuous variables. α = 0.05.

Urine parameters			
	NSF (N=193)	CaOx (N=309)	CaP (N=28)
Urine volume (l/24h)	2.10 ± 0.84	1.81 ± 0.77	2.04 ± 0.67
pH	6.04 ± 0.50	5.92 ± 0.51	5.97 ± 0.50
Bicarbonate (mmol/24h)	2.27 [0.886, 4.47]	1.36 [0.457, 3.57]	1.51 [1.06, 3.84]
Ammonium (mmol/24h)	28.70 ± 12.45	24.66 ± 11.84	25.63 ± 14.02
Citrate (mmol/24h)	3.39 ± 1.39	2.79 ± 1.35	2.63 ± 1.46
Sulfate (mmol/24h)	20.24 ± 9.00	18.72 ± 7.93, n = 174	18.56 ± 8.22, n = 14
Urea (mmol/24h)	369.91 ± 137.76	349.08 ± 148.77	353.83 ± 172.77
Sodium (mmol/24h)	154.60 ± 65.20	158.58 ± 71.38	159.49 ± 89.35
Potassium (mmol/24h)	74.95 ± 35.69	59.43 ± 26.30	57.93 ± 25.18
Chloride (mmol/24h)	141.82 ± 67.59	145.58 ± 66.69	182.68 ± 223.37
Calcium (mmol/24h)	4.15 [2.63, 5.71]	5.50 [3.69, 7.54]	5.43 [3.72, 9.42]
Inorganic phosphate (mmol/24h)	27.56 ± 11.19	26.97 ± 11.01	26.94 ± 14.76
Magnesium (mmol/24h)	4.13 ± 1.52	3.83 ± 1.67	3.72 ± 1.68
Oxalate (mmol/24h)	0.44 ± 0.28	0.33 ± 0.34	0.29 ± 0.14
Albumin (mg/24h)	2.90 [2.00, 5.46]	5.95 [2.77, 12.2], n = 307	8.80 [2.63, 16.6], n = 27
Protein (g/24h)	0.11 ± 0.04	0.12 ± 0.08, n = 308	0.14 ± 0.11
Creatinine (mmol/24h)	13.05 ± 4.48	12.77 ± 4.50	11.54 ± 4.44
Net acid excretion (mEq/24h)	42.50 ± 23.75	40.61 ± 21.06	41.26 ± 25.96

Supplementary Table 3. Urine parameters in SF and NSF individuals. Baseline urine data are shown as mean ± SD and continuous skewed data as median [25th percentile; 75th percentile]. Parameters with

missing data have their sample size (n) shown next to the mean or median value of each group. NAE = net acid excretion

CaOx unadjusted

Urine parameter	estimate	Std error	statistic	p-value	OR	2.50%	97.50%
(Intercept)	0.60	0.11	5.63	<0.001	1.83	1.48	2.27
Ammonium	-0.79	0.15	-5.17	<0.001	0.45	0.33	0.61
pH	-0.35	0.11	-3.20	0.001	0.70	0.56	0.87
Citrate	-0.51	0.12	-4.35	<0.001	0.60	0.47	0.75
Phosphate	0.20	0.15	1.33	0.184	1.23	0.91	1.66
Calcium	1.03	0.16	6.61	<0.001	2.81	2.09	3.87
Magnesium	-0.28	0.15	-1.91	0.056	0.75	0.56	1.00
Oxalate	-0.20	0.15	-1.33	0.185	0.82	0.60	1.09

Supplementary Table 4. Logistic regression model 0 with CaOx stone formation as dependent variable. OR = odds ratio. The p-values were obtained from a Wald test with $\alpha = 0.05$.

CaOx adjusted

Urine parameter	estimate	Std error	statistic	p-value	OR	2.50%	97.50%
(Intercept)	0.25	0.19	1.13	0.263	1.28	0.88	1.88
Ammonium	-0.84	0.16	-5.19	<0.001	0.43	0.31	0.59
pH	-0.25	0.12	-2.03	0.043	0.78	0.61	0.99
Citrate	-0.56	0.12	-4.53	<0.001	0.57	0.44	0.72
Phosphate	0.01	0.16	0.05	0.964	1.01	0.73	1.39
Calcium	1.05	0.16	6.60	<0.001	2.86	2.12	3.96
Magnesium	-0.22	0.15	-1.45	0.146	0.80	0.60	1.08
Oxalate	-0.15	0.15	-0.94	0.346	0.86	0.63	1.16
Age	0.05	0.12	0.45	0.942	1.06	0.84	1.34
BMI	0.49	0.12	3.96	<0.001	1.63	1.29	2.09
Sex (male)	0.61	0.25	2.45	0.014	1.84	1.13	3.15

Supplementary Table 5. Summary of results of the logistic regression model 1 with CaOx stone formation as dependent variable. OR = odds ratio. The p-values were obtained from a Wald test with $\alpha = 0.05$.

CaOx with AB score

Urine parameter	estimate	Std error	statistic	p-value	OR	2.50%	97.50%
(Intercept)	0.58	0.10	5.50	<0.001	1.78	1.45	2.19
AB score	-0.39	0.11	-3.57	<0.001	0.68	0.55	0.84
Citrate	-0.45	0.11	-3.94	<0.001	0.64	0.51	0.79
Phosphate	-0.05	0.14	-0.39	0.728	0.95	0.72	1.24
Calcium	0.94	0.15	6.17	<0.001	2.57	1.92	3.50
Magnesium	-0.31	0.14	-2.18	0.026	0.73	0.55	0.97
Oxalate	-0.32	0.15	-2.15	0.036	0.72	0.53	0.96

Supplementary Table 6. Logistic regression model 0 with CaOx stone formation as dependent variable and AB score replacing urine ammonium and pH. OR = odds ratio. The p-values were obtained from a Wald test with $\alpha = 0.05$.

CaOx with NAEC adjusted

Urine parameter	estimate	Std error	statistic	p-value	OR	2.50%	97.50%
(Intercept)	0.17	0.19	0.88	0.318	1.18	0.82	1.72
NAEC	-0.76	0.15	-4.98	<0.001	0.47	0.35	0.63
Citrate	-0.53	0.12	-4.43	<0.001	0.59	0.46	0.74
Calcium	1.06	0.16	6.68	<0.001	2.88	2.14	3.98
Magnesium	-0.20	0.14	-1.42	0.156	0.81	0.61	1.08
Oxalate	-0.22	0.15	-1.44	0.151	0.80	0.59	1.07
Age	-0.02	0.12	-0.17	0.870	1.02	0.81	1.28
BMI	0.51	0.12	4.14	<0.001	1.66	1.31	2.13
Sex (male)	0.73	0.24	2.97	0.003	2.06	1.28	3.33

Supplementary Table 7. Summary of results of the logistic regression model 1 with CaOx stone formation as dependent variable and NAEC replacing ammonium, pH, and phosphate. OR = odds ratio. The p-values were obtained from a Wald test with $\alpha = 0.05$.

CaOx with AB adjusted

Urine parameter	estimate	Std error	statistic	p-value	OR	2.50%	97.50%
(Intercept)	0.27	0.19	1.41	0.132	1.31	0.90	1.91
AB score	-0.31	0.12	-2.62	0.011	0.73	0.58	0.92
Citrate	-0.49	0.12	-4.07	<0.001	0.62	0.48	0.77
Phosphate	-0.27	0.15	-1.81	0.085	0.76	0.56	1.02
Calcium	0.95	0.16	6.06	<0.001	2.58	1.92	3.54
Magnesium	-0.27	0.15	-1.78	0.066	0.77	0.57	1.03
Oxalate	-0.29	0.15	-1.88	0.066	0.75	0.55	1.00
Age	0.08	0.12	0.69	0.410	1.09	0.87	1.36
BMI	0.44	0.12	3.67	<0.001	1.56	1.24	1.99
Sex (male)	0.52	0.24	2.15	0.038	1.69	1.05	2.73

Supplementary Table 8. Summary of results of the logistic regression model 1 with CaOx stone formation as dependent variable and NAEC replacing ammonium, pH, and phosphate. OR = odds ratio. The p-values were obtained from a Wald test with $\alpha = 0.05$.

ATC Code	Common name of the medication or supplement
A12BA02	Potassium citrate
A12BA04	Potassium Bicarbonate
A12CC04	Magnesium citrate
B05CB02	Sodium citrate
B05CB04	Sodium bicarbonate (irrigating solution)
B05XA02	Sodium bicarbonate (solution additive)
A02BC02	Pantoprazole
C03AA03	Hydrochlorothiazide
C09AA01	Captopril
C09AA02	Enalapril
C09AA03	Lisinopril
C09BA02	Enalapril and diuretics
C09BA03	Lisinopril and diuretics
C09BA05	Ramipril + hydrochlorothiazide
C09CA06	Candesartan
C09CA06	Candesartan + hydrochlorothiazide
C09CA08	Olmesartan medoxomil
C09DA01	Losartan and diuretics
C09DA03	Valsartan + hydrochlorothiazide
C09DA04	Ibesartan + hydrochlorothiazide
C09DA08	Olmesartan medoxomil and diuretics
C09DB02	Olmesartan medoxomil and amlodipine

C09DX03	Olmesartan medoxomil, amlodipine and hydrochlorothiazide
L04AD01	Cyclosporine
S01EC01	Acetazolamide

Supplementary Table 9. Medication and supplements used as exclusion criteria in subpopulations 1. ATC code = The Anatomical Therapeutic Chemical code.

Subpopulation after exclusion by supplement and drug intake

CaOx unadjusted

Urine parameter	estimate	Std error	statistic	p-value	OR	2.50%	97.50%
(Intercept)	0.09	0.12	0.770	0.441	1.09	0.87	1.38
Ammonium	-0.78	0.18	-4.430	<0.001	0.46	0.32	0.64
pH	-0.24	0.12	-1.908	0.056	0.79	0.62	1.00
Citrate	-0.39	0.14	-2.883	0.004	0.68	0.51	0.88
Phosphate	0.24	0.17	1.420	0.156	1.27	0.91	1.79
Calcium	1.02	0.17	5.929	<0.001	2.76	2.01	3.93
Magnesium	-0.29	0.17	-1.691	0.091	0.75	0.53	1.04
Oxalate	-0.58	0.19	-3.042	0.002	0.56	0.38	0.81

Supplementary Table 10. Summary of results of the logistic regression model 0 with CaOx stone formation as dependent variable in subpopulation 1. OR = odds ratio. The p-values were obtained from a Wald test with $\alpha = 0.05$.

CaOx with NAEC unadjusted

Urine parameter	estimate	Std error	statistic	p-value	OR	2.50%	97.50%
(Intercept)	0.10	0.12	0.838	0.402	1.10	0.88	1.38
NAEC	-0.48	0.15	-3.201	0.001	0.62	0.46	0.83
Citrate	-0.33	0.13	-2.532	0.011	0.72	0.56	0.92
Calcium	1.07	0.17	6.242	<0.001	2.92	2.11	4.14
Magnesium	-0.28	0.17	-1.689	0.091	0.75	0.54	1.04
Oxalate	-0.63	0.19	-3.350	0.001	0.53	0.37	0.76

Supplementary Table 11. Summary of results of the logistic regression model 0 with CaOx stone formation as dependent variable and NAEC replacing ammonium, pH, and phosphate in subpopulation 1. OR = odds ratio. The p-values were obtained from a Wald test with $\alpha = 0.05$.

CaOx adjusted

Urine parameter	estimate	Std error	statistic	p-value	OR	2.50%	97.50%
(Intercept)	-0.41	0.21	-1.93	0.054	0.66	0.43	1.00
Ammonium	-0.83	0.18	-4.52	<0.001	0.44	0.30	0.62
pH	-0.17	0.13	-1.26	0.210	0.84	0.65	1.10
Citrate	-0.38	0.14	-2.71	0.007	0.68	0.51	0.89
Phosphate	-0.01	0.18	-0.06	0.949	0.99	0.59	1.42
Calcium	1.07	0.17	6.19	<0.001	2.91	2.11	4.16
Magnesium	-0.25	0.18	-1.45	0.147	0.78	0.55	1.09
Oxalate	-0.56	0.20	-2.85	0.004	0.57	0.38	0.83
Age	-0.11	0.13	-0.85	0.396	0.89	0.69	1.16
BMI	0.46	0.14	3.36	0.001	1.58	1.21	2.07
Sex (male)	0.84	0.28	2.95	0.003	2.31	1.33	4.05

Supplementary Table 12. Summary of results of the logistic regression model 1 with CaOx stone formation as dependent variable in subpopulation 1. OR = odds ratio. The p-values were obtained from a Wald test with $\alpha = 0.05$.

CaOx with NAEC adjusted

Urine parameter	estimate	Std error	statistic	p-value	OR	2.50%	97.50%
(Intercept)	-0.45	0.21	-2.14	0.033	0.64	0.42	0.96
NAEC	-0.70	0.17	-4.21	<0.001	0.50	0.35	0.68
Citrate	-0.33	0.13	-2.47	0.013	0.72	0.55	0.93
Calcium	1.06	0.17	6.15	<0.001	2.88	2.09	4.10
Magnesium	-0.28	0.17	-1.64	0.101	0.76	0.54	1.05
Oxalate	-0.61	0.20	-3.12	0.002	0.55	0.37	0.79
Age	-0.15	0.13	-1.18	0.239	0.86	0.66	1.11
BMI	0.46	0.13	3.47	0.001	1.59	1.23	2.08
Sex (male)	0.90	0.27	3.29	0.001	2.47	1.45	4.25

Supplementary Table 13. Summary of results of the logistic regression model 1 with CaOx stone formation as dependent variable and NAEC replacing ammonium, pH, and phosphate in subpopulation 1. OR = odds ratio. The p-values were obtained from a Wald test with $\alpha = 0.05$.

CaOx with AB unadjusted

Urine parameter	estimate	Std error	statistic	p-value	OR	2.50%	97.50%
(Intercept)	0.08	0.12	0.70	0.486	1.08	0.86	1.36
AB score	-0.25	0.12	-2.09	0.037	0.78	0.61	0.98
Citrate	-0.32	0.13	-2.44	0.015	0.73	0.56	0.93
Phosphate	-0.03	0.15	-0.18	0.860	0.98	0.72	1.31
Calcium	0.95	0.17	5.51	<0.001	2.58	1.86	3.66
Magnesium	-0.36	0.17	-2.13	0.033	0.70	0.50	0.97
Oxalate	-0.69	0.19	-3.62	<0.001	0.50	0.34	0.72

Supplementary Table 14. Summary of results of the logistic regression model 0 with CaOx stone formation as dependent variable and AB score replacing ammonium and pH in subpopulation 1. OR = odds ratio. The p-values were obtained from a Wald test with $\alpha = 0.05$.

CaOx with AB adjusted

Urine parameter	estimate	Std error	statistic	p-value	OR	2.50%	97.50%
(Intercept)	-0.38	0.21	-1.78	0.075	0.68	0.45	1.04
AB score	-0.21	0.13	-1.57	0.116	0.81	0.63	1.05
Citrate	-0.30	0.14	-2.25	0.024	0.74	0.56	0.95
Phosphate	-0.30	0.17	-1.76	0.078	0.74	0.53	1.03
Calcium	0.97	0.17	5.55	<0.001	2.64	1.90	3.77
Magnesium	-0.33	0.17	-1.89	0.059	0.72	0.51	1.00
Oxalate	-0.69	0.20	-3.48	0.001	0.50	0.34	0.73
Age	-0.08	0.13	-0.62	0.535	0.92	0.71	1.19
BMI	0.42	0.13	3.16	0.002	1.52	1.18	1.98
Sex (male)	0.76	0.28	2.74	0.006	2.13	1.24	3.69

Supplementary Table 15. Summary of results of the logistic regression model 1 with CaOx stone formation as dependent variable and AB score replacing ammonium and pH in subpopulation 1. OR = odds ratio. The p-values were obtained from a Wald test with $\alpha = 0.05$.

Models with GI alkali absorption without exclusion by supplement and drug intake

CaOx with GI alkali absorption unadjusted

Urine parameter	estimate	Std error	statistic	p-value	OR	2.50%	97.50%
(Intercept)	0.51	0.10	5.17	< 0.001	1.66	1.37	2.02
GI alkali absorption	-0.31	0.13	-2.42	0.016	0.74	0.56	0.93
Ammonium	-0.35	0.11	-3.11	0.002	0.70	0.56	0.88
pH	-0.23	0.11	-2.12	0.034	0.80	0.65	0.98
Citrate	-0.25	0.11	-2.38	0.017	0.78	0.63	0.96
Oxalate	-0.28	0.13	-2.14	0.032	0.75	0.58	0.97

Supplementary Table 16. Summary of results of the logistic regression model 0 with CaOx stone formation as dependent variable and with GI alkali absorption replacing calcium, magnesium, and phosphate. OR = odds ratio. The p-values were obtained from a Wald test with α = 0.05.

CaOx with GI alkali absorption adjusted

Urine parameter	estimate	Std error	statistic	p-value	OR	2.50%	97.50%
(Intercept)	0.05	0.18	0.27	0.788	1.05	0.74	1.49
GI alkali absorption	-0.32	0.14	-2.29	0.022	0.73	0.55	0.94
Ammonium	-0.47	0.13	-3.74	<0.001	0.62	0.48	0.80
pH	-0.11	0.12	-0.90	0.366	0.90	0.71	1.13
Citrate	-0.32	0.11	-2.85	0.004	0.73	0.58	0.90
Oxalate	-0.24	0.13	-1.83	0.067	0.78	0.59	1.01
Age	0.10	0.12	0.87	0.382	1.11	0.88	1.39
BMI	0.43	0.11	3.73	<0.001	1.53	1.23	1.93
Sex (male)	0.75	0.23	3.33	0.001	2.13	1.37	3.32

Supplementary Table 17. Summary of results of the logistic regression model 1 with CaOx stone formation as dependent variable and GI alkali absorption replacing a calcium, magnesium, and phosphate. OR = odds ratio. The p-values were obtained from a Wald test with $\alpha = 0.05$.
